# Intraperitoneal immunotherapy with T cells stably and transiently expressing anti-EpCAM CAR in xenograft models of peritoneal carcinomatosis

**DOI:** 10.18632/oncotarget.14592

**Published:** 2017-01-10

**Authors:** Wei Xia Ang, Zhendong Li, Zhixia Chi, Shou-Hui Du, Can Chen, Johan C.K. Tay, Han Chong Toh, John E. Connolly, Xue Hu Xu, Shu Wang

**Affiliations:** ^1^ Department of Biological Sciences, National University of Singapore 117543, Singapore; ^2^ Institute of Bioengineering and Nanotechnology 138669, Singapore; ^3^ Tessa Therapeutics, Pte Ltd., 239351, Singapore; ^4^ Division of Medical Oncology, National Cancer Centre, 169610, Singapore; ^5^ Programme in Translational Immunology, Institute for Molecular and Cell Biology 138648, Singapore; ^6^ Department of Gastrointestinal Surgery, The Third Affiliated Hospital of Guangzhou Medical University, Guangzhou 510150, China

**Keywords:** peritoneal carcinomatosis, EpCAM, chimeric antigen receptor, T lymphocytes, adoptive immunotherapy

## Abstract

The epithelial cell adhesion molecule (EpCAM) is overexpressed in a wide variety of tumor types, including peritoneal carcinomatosis (PC) from gastrointestinal and gynecological malignancies. To develop a chimeric antigen receptor T (CART) cell therapy approach to treat patients with end-stage PC, we constructed third generation CARs specific to EpCAM using the 4D5MOC-B single chain variable fragment. CART cells were generated with lentiviral transduction and exhibited specific *in vitro* killing activity against EpCAM-positive human ovarian and colorectal cancer cells. A single intraperitoneal injection of the CART cells eradicated established ovarian xenografts and resulted in significantly prolonged animal survival. Since EpCAM is also expressed on normal epithelium, anti-EpCAM CART cells were generated by mRNA electroporation that display a controlled cytolytic activity with a limited CAR expression duration. Multiple repeated infusions of these RNA CAR-modified T cells delayed disease progression in immunodeficient mice bearing well-established peritoneal ovarian and colorectal xenografts. Thus, our study demonstrates the effectiveness of using anti-EpCAM CAR-expressing T cells for local treatment of PC in mice. The possibility of using this approach for clinical treatment of EpCAM-positive gastrointestinal and gynecological malignancies warrants further validation.

## INTRODUCTION

The epithelial cell adhesion molecule CD326 (EpCAM), a 40-kDa transmembrane glycoprotein, has been a favorable therapeutic target for anticancer strategies because of its tumor-specific overexpression [1–4]. In normal tissues, the expression of EpCAM is restricted to simple epithelia, where it is expressed only on the basolateral cell surface, thus being apically inaccessible [5]. EpCAM is upregulated in a variety of epithelial-derived carcinomas, including adenocarcinomas of colon, stomach, pancreas, lung, ovarian, and breast (≥ 90%)[6], and functions as a potent signal transducer that can be activated by regulated intramembrane proteolysis to display oncogenic effects through the Wnt pathway [7]. In cancer tissue, the subcellular distribution of EpCAM is changed from the basolateral location to a homogeneously dispersed pattern on cancer cell surface, making the molecule easily accessible to cellular and antibody-based immunotherapy [1, 3, 4, 8–10]

Taking the feature that the inner layer of the peritoneum consists of EpCAM-negative mesothelial cells, anti-EpCAM antibody therapeutics have been applied to the peritoneal cavity to treat patients with peritoneal carcinomatosis (PC). Catumaxomab (Removab), a trifunctional anti-EpCAM/anti-CD3 antibody, has been tested in an open label, multicenter, randomized phase II/III trial in 258 patients with malignant ascites [11]. Intraperitoneal infusions of catumaxomab, preceded by aspiration of ascites, significantly prolonged puncture-free survival, although median overall survival was similar between the two groups: 72 days vs 68 days [11]. Based on this result, an approval for intraperitoneal infusion of catumaxomab was granted by the European Union in April 2009 [12]. Two years later, a phase I/II trial using catumaxomab immunotherapy to treat PC in patients with non-ascites-accumulating and non-resectable PC from colon, gastric, or pancreatic cancer reported median overall survival of 502 days for the catumaxomab group, which compared very favorably with their control group (6 months) and with the previous literature [13]. In 2014, a phase II study of catumaxomab in heavily pretreated patients with chemotherapy-refractory ovarian cancer and recurrent symptomatic malignant ascites has demonstrated a 2-fold prolonged median puncture-free interval and 111 days of median overall survival [14]. Moreover, recombinant anti-EpCAM immunotoxins have been tested in clinical trials by giving intratumorally in head and neck cancer [15], intravesically in patients with bladder cancer [16] and intravenously in patients with metastatic disease [17].

Given the varying levels of success as well as acceptable safety profile yielded by anti-EpCAM antibody therapeutics, anti-EpCAM chimeric antigen receptors (CAR) have been developed to modify natural killer (NK) cells and T cells to kill EpCAM-expressing prostate cancer cells and breast carcinoma cells [18–24]. We sought to develop a CART cell technology to target EpCAM-positive cancers for patients with end-stage peritoneal metastasis who otherwise have no viable treatment options. We here report the development of a potent third-generation anti-EpCAM CAR using a well-studied humanized monoclonal antibody. We characterize the CAR effects in peritoneal dissemination mouse models of human ovarian cancer and human colorectal cancer. The mouse models were established by injecting SKOV3-Luc, a human ovarian cancer cell line, and HRT-18G, a human colorectal cancer cell line, into the peritoneal cavity of immunodeficient NSG mice. These models displayed a phenotype similar to late stage metastatic cancers in patients, including extensive peritoneal metastasis and ascites production. Our results demonstrate that immunotherapy with anti-EpCAM CAR-expressing T cells, either modified with lentiviral transduction or mRNA electroporation, can effectively suppress peritoneal tumor progression and, therefore, could potentially be used to treat late-stage PC.

## RESULTS

### Generation of T cells stably expressing anti-EpCAM CAR using lentiviral transduction and artificial antigen-presenting cells

**Figure 1:**
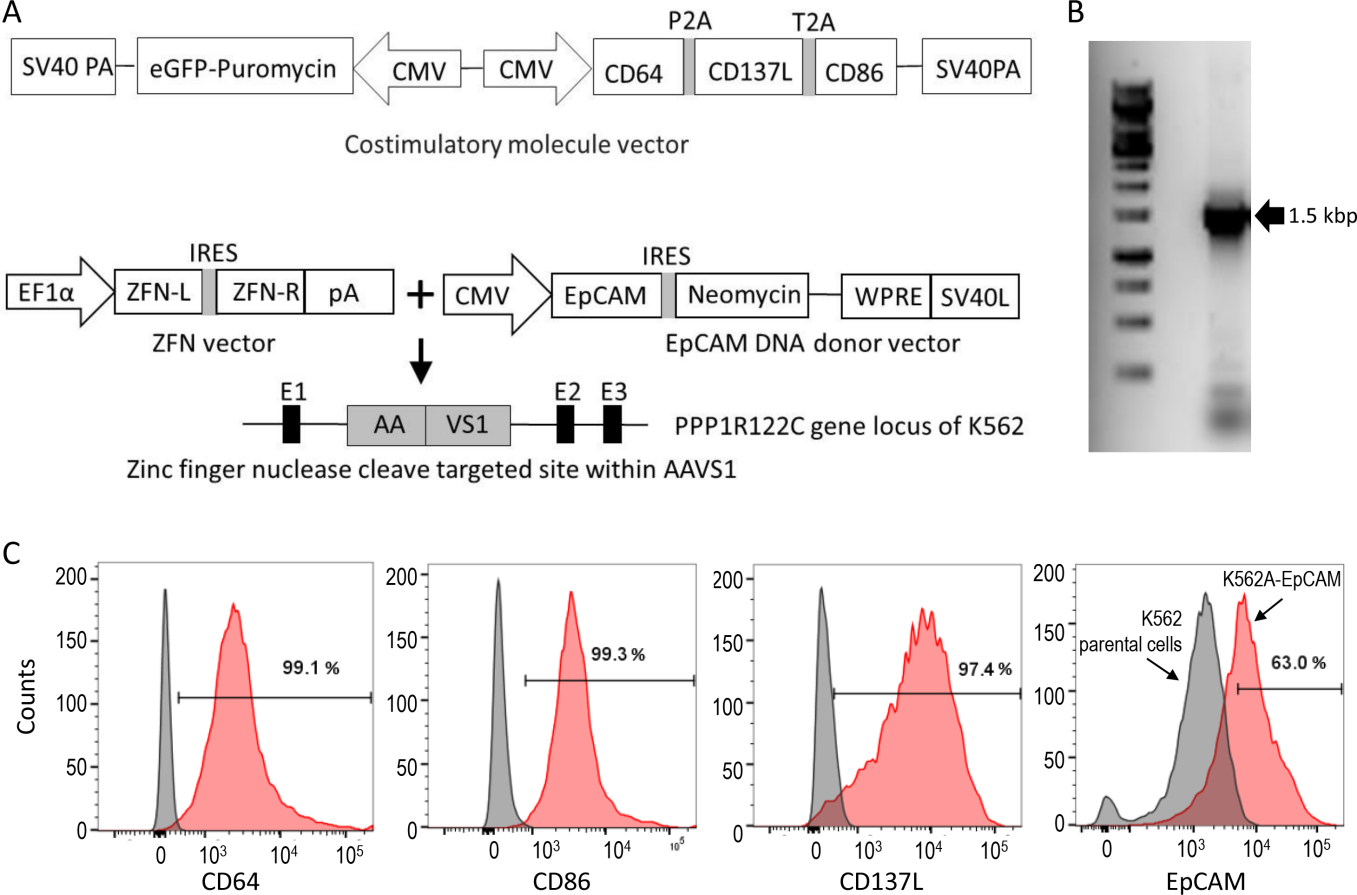
Preparation of a K562 aAPC line for expansion of anti-EpCAM CAR-expressing T cells (**A**) Schematic diagrams of plasmid vectors used for the generation of the aAPC cell line. The costimulatory molecule vector (top) was used for transfection of wild-type K562 cells to generate puromycin-resistant K562 cells expressing CD64, CD137L and CD86 (K562A). K562A cells were further modified by co-transfection with the ZFN vector and EpCAM DNA donor vector (bottom) for AAVS1 locus-specific gene insertion to generate puromycin- and neomycin-resistant aAPCs expressing EpCAM (K562A-EpCAM). (**B**) PCR genome typing to demonstrate the AAVS1 locus-specific gene insertion of the EpCAM gene, as indicated by the presence of one single 1.5-kb band. (**C**) Phenotype analysis of K562A-EpCAM cells. Flow cytometric analysis demonstrates the surface expression of CD64, CD86, CD137L, and EpCAM. In the panels for CD64, CD86, and CD137L, left curves: isotype controls; right curves: antibodies. In the panel for EpCAM, left curve: K562 parental cells; right curve: K562A-EpCAM cells.

T cells can be readily modified for CAR expression with retroviral or lentiviral vectors. However, large-scale production of viral vectors for the clinical manufacture of CAR-expressing T cells is not trivial, and can be expensive. After viral transduction, enrichment of CAR-expressing T cells by selectively propagating stably modified cells may facilitate the production of cell therapeutics with improved purity and potency. To enrich T cells stably modified with a lentiviral vector containing an anti-EpCAM CAR gene, we developed a K562-based artificial antigen-presenting cell (aAPC) line for numeric expansion of the modified T cells in a CAR-mediated manner. A costimulatory molecule vector (Figure 1A) containing CD64, CD137L, CD86, and puromycin genes was constructed and used for transfection of wild-type K562 cells. The generated puromycin-resistant K562 cells were further modified with the zinc-finger nuclease technology for AAVS1 locus-specific insertion of the EpCAM gene together with the neomycin gene. Single cell cloning of the puromycin- and neomycin-resistant K562 cells was performed. After PCR genome typing to select the clones with the AAVS1 locus-specific gene insertion of the EpCAM gene (Figure 1B) and flow cytometric analysis of the surface expression of CD64, CD86, CD137L and EpCAM (Figure 1C), one clone, named K562A-EpCAM, was selected for subsequent experiments.

**Figure 2:**
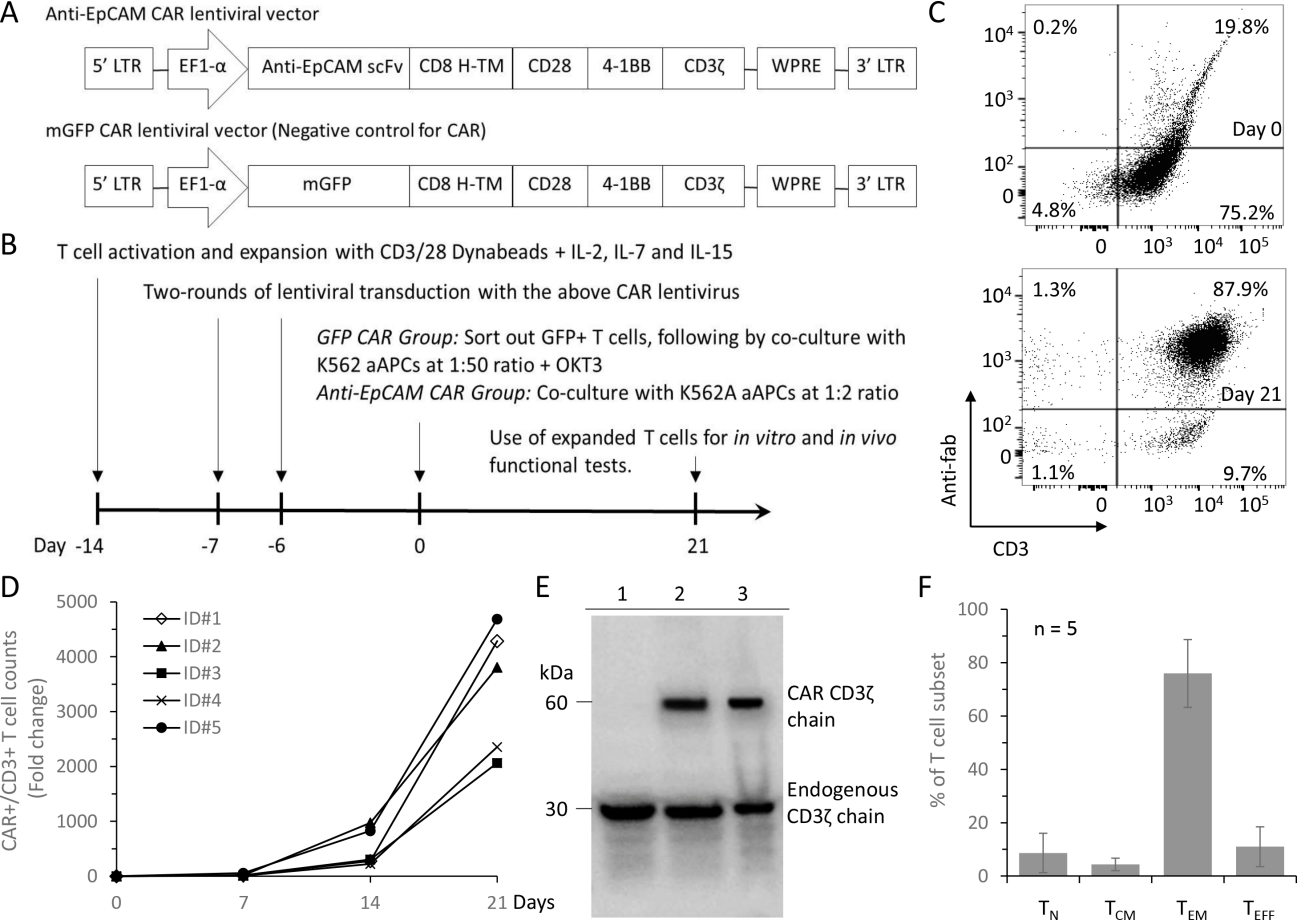
Generation and expansion of EpCAM-specific CART cells (**A**) Schematics of lentiviral vectors used in the study. The scFv region that recognizes EpCAM was derived from 4D5MOC-B humanized mAb. Anti-EpCAM CAR contains the CD28 and 4-1BB co-stimulatory domains and the CD3zeta T cell activation domain. mGFP CAR control vector was constructed using the GFP sequence instead of EpCAM-specific scFv. (**B**) Schematic illustration of EpCAM-specific CART cell generation and expansion. (**C**) Flow cytometric analysis to detect the surface expression of anti-EpCAM CAR on the genetically modified T cells before and after 21 days of co-culturing with K562A-EpCAM aAPCs using anti-mouse IgG Fab and anti-human CD3 antibodies. Representative FACS plots are shown. (**D**) Increase in the number of CD3+CAR+ T cells over the co-culturing with K562A-EpCAM cells. The number of initially seeded T cells was 2E4 per well. Results from five different PBMC samples are shown. (**E**) Western blot analysis using a CD3ζ-specific antibody confirms the CAR expression in modified T cells 21 days after co-culture with K562A-EpCAM cells. The endogenous CD3ζ was stained as an internal loading control. Lane 1: unmodified T cells; Lane 2: T cells transduced with mGFP CAR lentiviral vectors; Lane 3: T cells transduced with anti-EpCAM CAR lentiviral vectors. (**F**) Phenotyping of the propagated EpCAM-specific CART cells. T cells collected after 21 days of co-culture with K562A-EpCAM cells were assessed by flow cytometry. T cells were gated for the presence of naive (T_N_, CCR7+CD45RA+), central memory (T_CM_, CCR7+CD45RA-), effector memory (T_EM_, CCR7-CD45RA-), and terminally differentiated effector T cells (T_EFF_, CCR7-CD45RA+). Results from five different PBMC samples are shown.

To generate EpCAM-specific CART cells, we constructed a lentiviral transfer plasmid encoding a third-generation CAR composed of the single-chain variable fragment (scFv) from 4D5MOC-B humanized mAb [25], the CD8 alpha hinge region, CD28 co-stimulatory domain, 4-1BB co-stimulatory domain, and the CD3zeta T cell activation domain (Figure 2A). A control lentiviral plasmid was constructed by replacing the anti-EpCAM scFv sequence with the green fluorescent protein (GFP) coding sequence and named membrane-bound GFP (mGFP) CAR. The viruses encoding the anti-EpCAM CAR were used to transduce human CD3/CD28 dynabead-activated T cells and the transduced T cells expressing the CAR were selectively expanded by co-culturing with gamma-irradiated K562A-EpCAM aAPCs at a ratio of 1:2 for 3 weeks (Figure 2B). The percentage of anti-EpCAM CAR-expressing T cells was 16.9 ± 3.9% before co-culturing with the aAPCs, and the percentage increased to 84.7 ± 7.7% after the co-culturing for 3 weeks as revealed by flow cytometry using an Fc-specific antibody (Figure 2C and Supplementary Figure 1), representing an average 3440–fold numeric expansion of CD3+ CAR+ T cells (Figure 2D). The CAR expression in the expanded T cells was further analyzed in Western blots probed with anti-human CD3ζ. In T cells transduced with anti-EpCAM CAR and mGFP CAR lentiviruses, the expression of a 60-kDa chimeric CD3ζ product and the endogenous CD3ζ was confirmed, whereas untransduced T cells were stained only for the endogenous CD3ζ (Figure 2E). Band signal quantitation revealed that the ratio of CAR to endogenous CD3ζ ranged from 0.7 to 0.9. Retention of memory markers on subpopulations of the expanded EpCAM-specific CART cells was confirmed by flow cytometry. T cells gated for the presence of naive (T_N_, CCR7+CD45RA+), central memory (T_CM_, CCR7+CD45RA-), effector memory (T_EM_, CCR7-CD45RA-), and terminally differentiated effector T cells (T_EFF_, CCR7-CD45RA+) were 8.7%, 4.3%, 76.0% and 11.0% respectively (Figure 2F).

### Specific *in vitro* killing of EpCAM-positive tumor cells with T cells stably expressing anti-EpCAM CAR

**Figure 3:**
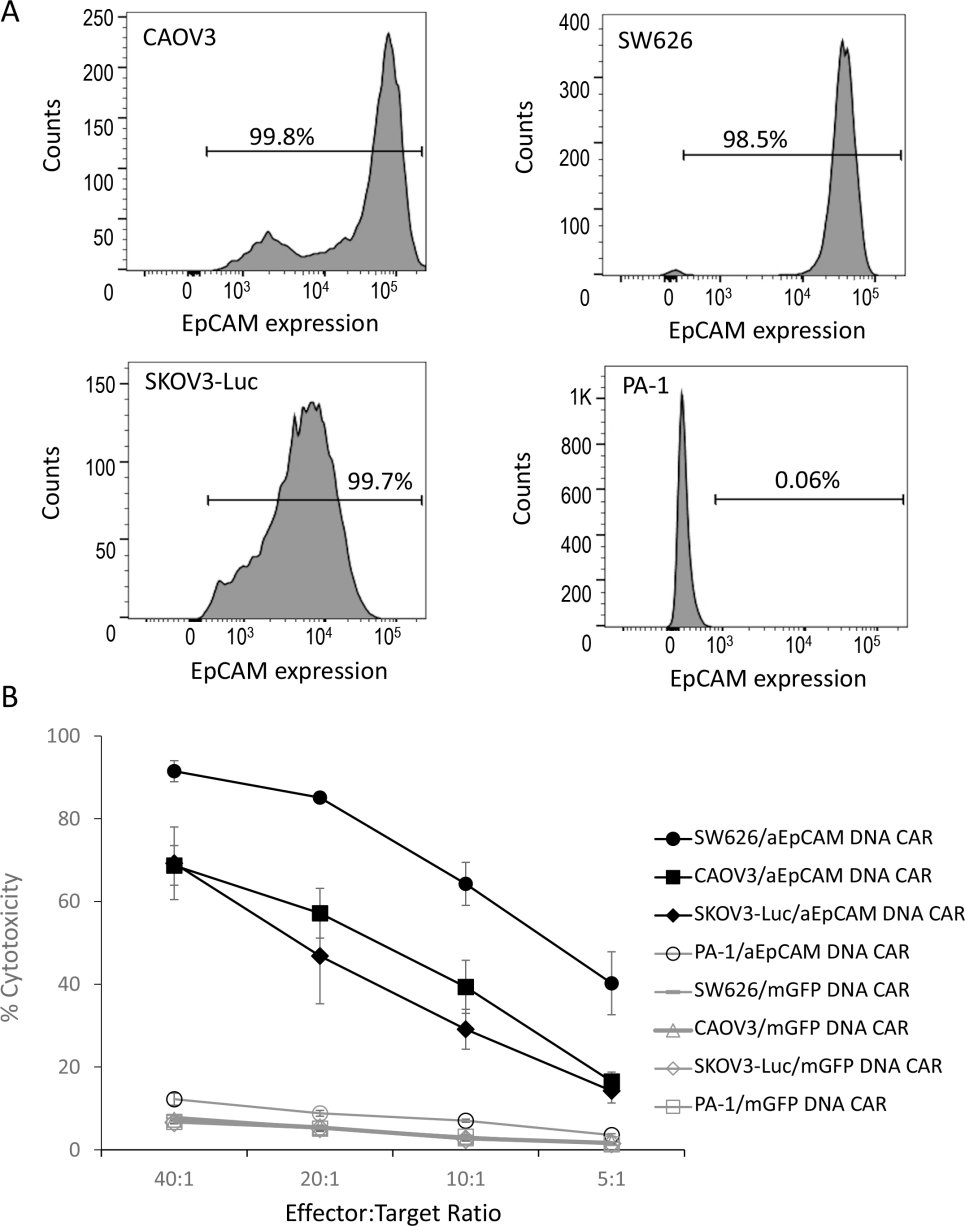
*In vitro* cell lysis of EpCAM-positive tumour cells with T cells genetically modified by a lentiviral anti-EpCAM CAR vector (**A**) EpCAM expression on ovarian cancer cells as demonstrated by flow cytometric analysis. Three EpCAM-positive human epithelial ovarian cancer cell lines (CAOV3, SW626, and SKOV3-luc) and one EpCAM-negative human ovarian cancer cell line (PA-1) were analysed. (**B**) % cytotoxicity. Delfia EuTDA cytotoxicity assay (3 hours EuTDA culturing) was used to assess the cytotoxicity of anti-EpCAM CAR-expressing T cells against EpCAM-positive ovarian cancer cell lines. Specific cell lysis was demonstrated by including EpCAM-negative PA-1 cells and the use of mGFP CAR. Mean ± SD of three validation runs is represented.

We then tested the enriched T cells stably expressing anti-EpCAM CAR for their anti-tumor cytotoxicity against human ovarian cancer cells. The expression of EpCAM on the surface of four human ovarian cancer cell lines, CAOV3, SW626, SKOV3-Luc, and PA-1, were examined with flow cytometry. High levels of EpCAM expression were observed in CAOV3, SW626, and SKOV3-Luc, whereas no EpCAM expression was detected on PA-1 (Figure 3A). The T cells stably expressing anti-EpCAM CAR displayed a high cell lysis activity towards EpCAM-positive ovarian cancer cells, being able to kill 69.2 ± 8.8% of SKOV3-Luc tumor cells, 68.7 ± 4.8% of CAOV-3 cells, and 91.5 ± 2.6% SW626 cells at an effector to target (E:T) ratio of 40:1 (Figure 3B). EpCAM-negative PA-1 cells were insensitive to anti-EpCAM CAR-expressing T cells: there were only 12.2 ± 1.5% cell death at E:T ratio of 40:1 (Figure 3B). The results indicate the specific recognition and killing of EpCAM-positive target cells by the enriched anti-EpCAM CAR-expressing T cells.

### T cells stably expressing anti-EpCAM CAR display tumor killing effects *in vivo*

**Figure 4:**
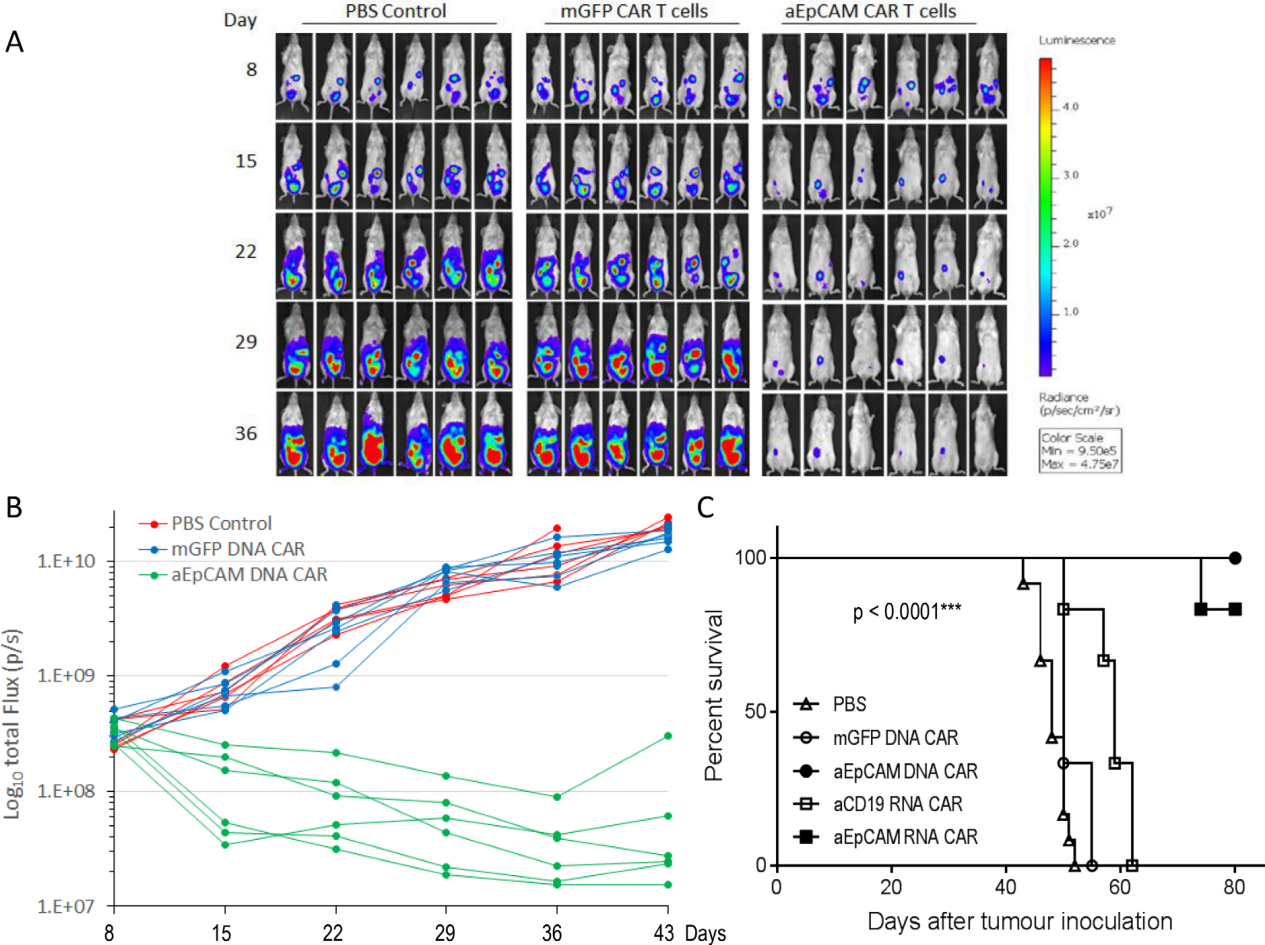
T cells genetically modified with a lentiviral anti-EpCAM CAR vector effectively treat established ovarian tumours in NSG mice NSG mice were i.p. injected with 1 × 10^7^ SKOV3-Luc ovarian cancer cells on day 0. The mice were randomized into three groups (*n* = 6 per group) before beginning CART cell treatment on day 8. Mice with disseminated ovarian tumours were given a single dose of 1 × 10^7^ CAR T cells (i.p.) or PBS. (**A**) Bioluminescent images prior to treatment (day 8) and following the treatments (day 15, 22, 29, and 36). (**B**) Tumor burden over time by bioluminescent imaging. Each mouse is represented by one line. The inhibition of tumour growth by the anti-EpCAM CAR-modified T cells maintained for at least 43 days in most of the treated mice. (**C**) Kaplan-Meier analysis of survival. For comparison purpose, survival results from Figure 7A and 7B are also shown here. The PBS curve represents the composite survival rate of two independent experiments (*n* = 12 totally). Statistical analysis of survival data was performed using the log-rank test.

We moved on to test *in vivo* tumor killing effects of the T cells stably expressing anti-EpCAM CAR. Ovarian cancer, due to its tendency to confine to the peritoneal cavity, provides a good model to test the regional delivery of CART cells therapy. We established a mouse ovarian cancer model in immunocompromised NSG mice by intraperitoneal (i.p.) injection of SKOV3-Luc cells. This ovarian cancer cell line contains a stably integrated firefly luciferase reporter gene that can be used for easily monitoring therapeutic effects with non-invasive *in vivo* imaging. Tumor progression was monitored by whole-body bioluminescence imaging of SKOV3-Luc cells (Figure 4A). On day 8 post-tumor inoculation, when all mice had established tumors in the peritoneal cavity, the animals were randomly divided into 3 groups (6 animals each) for treatment: group 1 was subjected to one i.p. injection of PBS, group 2 to one i.p. injection of T cells expressing mGFP CAR, and group 3 received one i.p. injection of the T cells stably expressing anti-EpCAM CAR. As shown in Figure 4B, the bioluminescence intensities, which are indicative of tumor burdens, in the PBS and mGFP CAR groups progressively increased from day 8 to day 43, demonstrating a rapid tumor progression after SKOV3-Luc inoculation, whereas the bioluminescence intensities in the anti-EpCAM CAR group quickly decreased after the treatment and remained low in most of the treated mice for at least 43 days. Attributed to the robust inhibitory effect of T cells stably expressing anti-EpCAM CAR on tumor growth, the survival of tumor-bearing mice in the anti-EpCAM CAR group was significantly improved. All mice treated with T cells stably expressing anti-EpCAM CAR survived for longer than 80 days, while all mice in the two control groups had died or had to be euthanized due to being moribund by day 55 (Figure 4C).

### *In vitro* effects of T cells electroporated with mRNA encoding anti-EpCAM CAR

**Figure 5:**
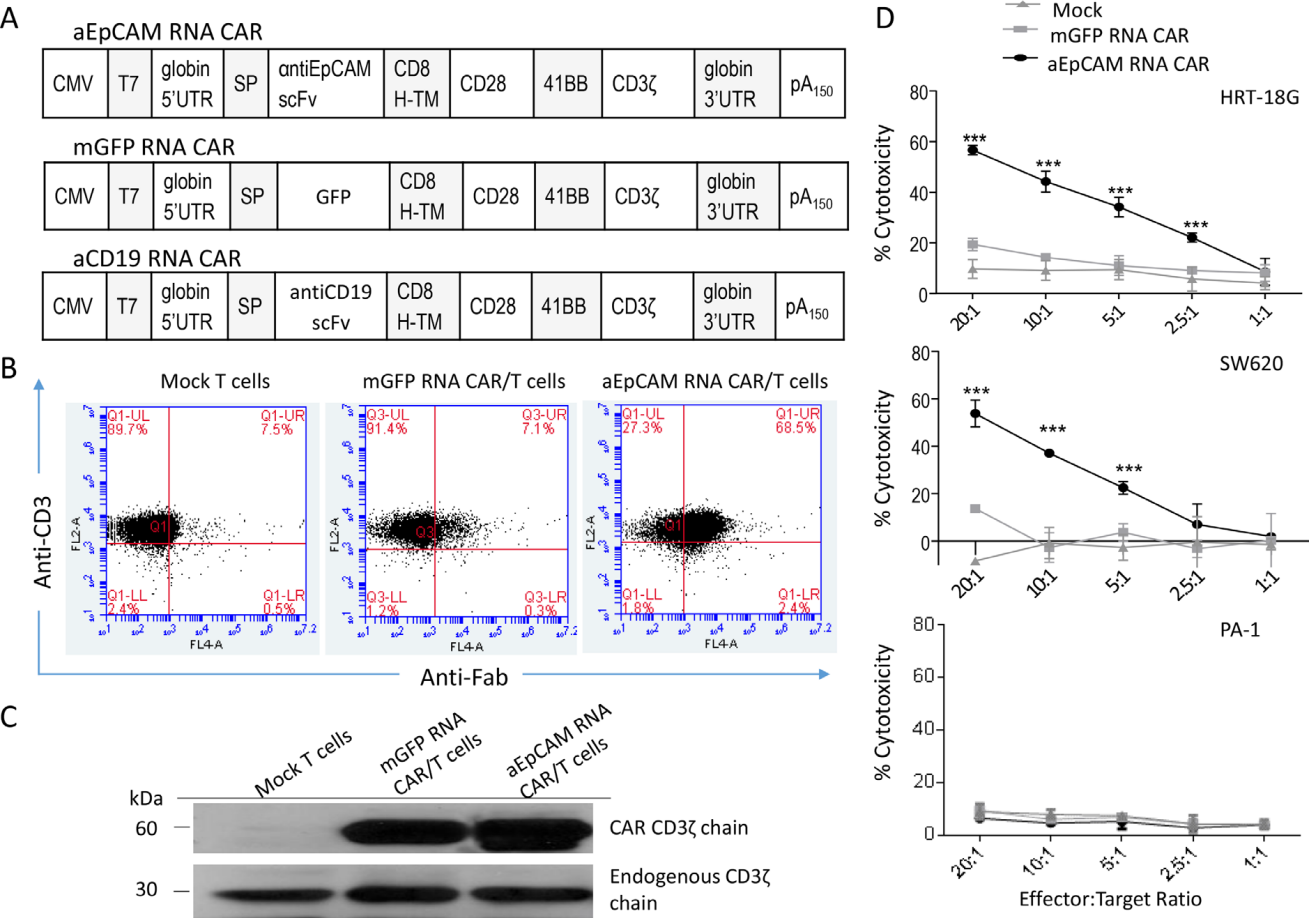
Electroporation of mRNA encoding anti-EpCAM CAR in human T cells and cytotoxicity of the CAR-modified T cells (**A**) Schematic diagrams of mRNA CAR constructs used in this study. (**B**) Flow cytometric analysis to examine anti-EpCAM CAR expression on T cells after electroporation. The cells were collected 24 hours after electroporation for analysis. (**C**) Western blot analysis using a CD3ζ-specific antibody confirms the CAR expression in T cells. The cells were collected 24 hours after electroporation for analysis. The endogenous CD3ζ was stained as an internal loading control. (**D**) *In vitro* cell lysis. Delfia EuTDA cytotoxicity assay (3 hours EuTDA culturing) was used to assess the cytotoxicity of anti-EpCAM RNA CAR-modified T cells against EpCAM-positive human cancer cell lines HRT-18G and SW620, as well as EpCAM-negative ovarian cancer cell line PA-1. mGFP RNA CAR-modified T cells were included as a control. Mean ± SD of three validation runs is represented. ***p < 0.001.

As EpCAM is expressed on normal epithelium, it is important to test T cells transfected with mRNA encoding anti-EpCAM CAR to provide self-limited expression of the CAR, which is useful to screen for immediate toxicity in a clinical trial. We constructed a plasmid vector to prepare mRNA encoding a third-generation CAR similar to the above described EpCAM-specific CAR-expressing lentiviral vector, together with two control CAR constructs, mGFP CAR and irrelevant anti-CD19 CAR (Figure 5A). Human T cell transfection with mRNA electroporation, after optimization with GFP mRNA, provided 97% efficiency (Supplementary Figure 2). The electroporation of T cells with mGFP RNA CAR gave 79% transfection efficiency (Supplementary Figure 2). Electroporation of mRNA encoding anti-EpCAM CAR (Figure 5A) in human T cells was then assessed by flow cytometric analysis with an Fc-specific antibody. High levels of surface CAR expression (up to 68.5%) were detectable (Figure 5B). To further confirm that CAR constructs expressed in T cells, Western blotting was performed using an anti-CD3zeta antibody. While only the endogenous CD3zeta chain was detected in un-transfected T cells, both the exogenous and endogenous CD3 zeta chains were detected in T cells transfected with anti-EpCAM RNA CAR and mGFP RNA CAR (Figure 5C).

EpCAM is highly expressed in most of gastrointestinal tumors and in some carcinomas of the genitourinary tract, but not in most soft-tissue tumors and all lymphomas [26]. We examined EpCAM expression in a panel of established human cell lines including lymphoma line Raji, melanoma line IGR1, colorectal cancer lines HCT8, HRT-18G, SW480, and SW620, and ovarian cancer lines PA-1, SKOV3-Luc, SW626, and CAOV-3. While Raji, IGR1, and PA-1 were EpCAM-negative, all other cell lines expressed high levels of EpCAM (Supplementary Figure 3). To evaluate the lytic specificity of T cells transfected with anti-EpCAM RNA CAR, HRT-18G, SW626 and PA-1 tumor cells were used as the targets in the Delfia EuTDA cytotoxicity assays. The killing against EpCAM-positive HRT-18G and SW626 cells by the RNA CAR-modified T cells significantly increased to close to 60% from the less than 20% cytotoxicity observed for the mock and mGFP RNA CAR controls at E:T ratio of 20:1, whereas the killing efficiency in EpCAM-negative PA-1 cells was similar to the control levels (Figure 5D), demonstrating a good specificity of anti-EpCAM RNA CAR-modified T cells in recognizing and killing EpCAM-positive target cells.

**Figure 6:**
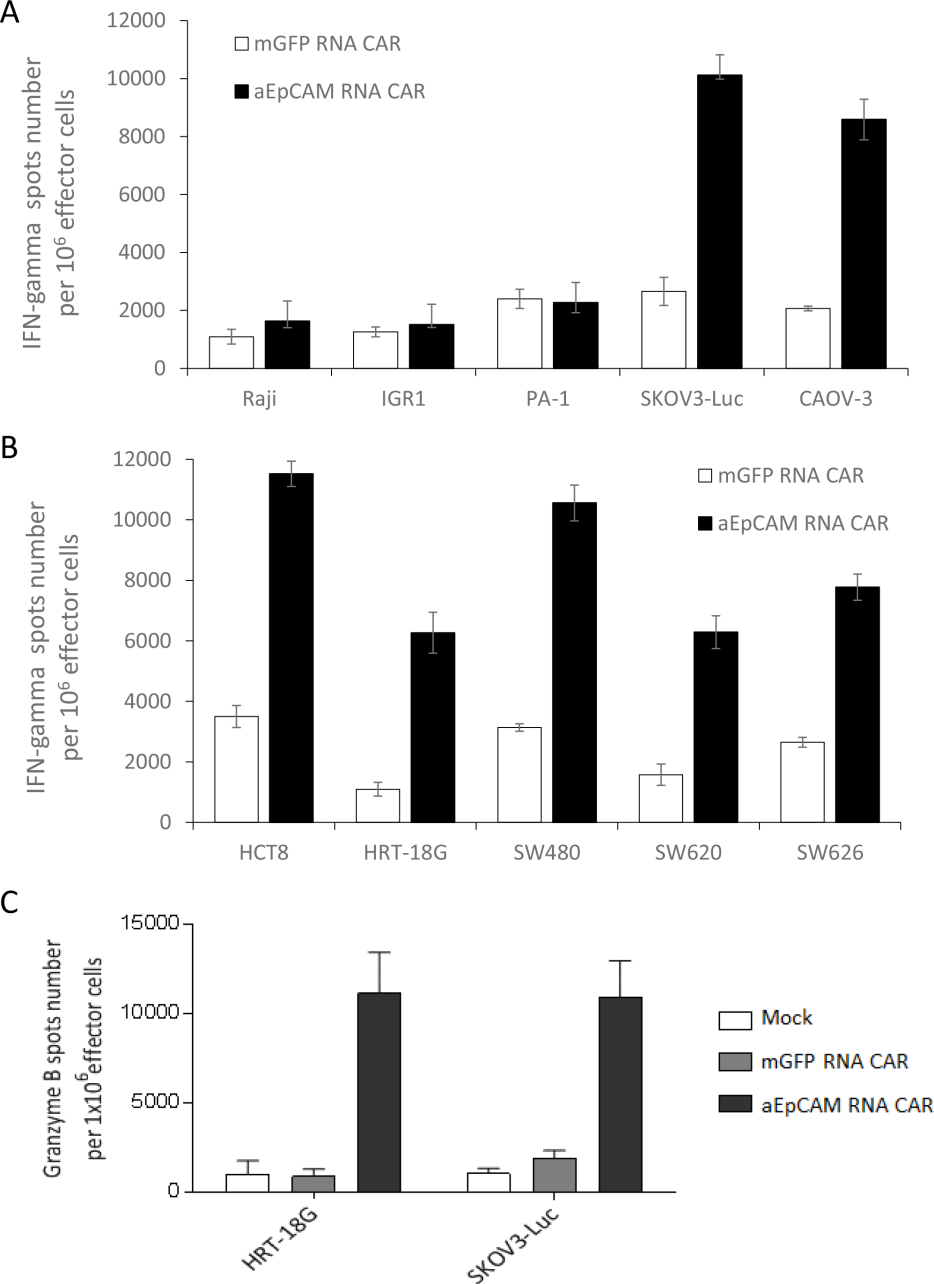
IFNγ secretion and granzyme B up-regulation triggered by tumor antigen-specific recognition of anti-EpCAM RNA CARs (**A** and **B**) Increased IFNγ secretion as determined by IFNγ ELISPOT assay. T cells were electroporated with anti-EpCAM RNA CARs and co-cultured with target tumor cells overnight before assay. mGFP RNA CAR-transfected T cells served as a negative control. Results from EpCAM-negative tumor cell lines Raji, IGR1, and PA-1 and EpCAM-positive cancer cell lines SKOV3-Luc and CAOV-3 are shown in (**A**) and results from EpCAM-positive cancer cell lines HCT8, HRT-18G, SW480, SW620 and SW626 in (**B**). (**C**) Granzyme B up-regulation as determined by granzyme B ELISPOT assay. EpCAM-positive tumor cell lines HRT-18G and SKOV3-Luc were tested as described in (A and B) Mean IFNγ or granzyme B spots per 1 × 10^6^ T cells ± SD from triplicate cultures are shown in A, B and C.

To further evaluate tumor-targeting efficiency, T cells transfected with anti-EpCAM RNA CAR or mGFP RNA CAR were co-cultured with above tumor cell lines and IFNγ secretion was determined by ELISPOT assay since Th-1 cytokine IFNγ secretion is associated with antitumor activity of T cells for adoptive immunotherapy. The scanned ELISPOT plates of EpCAM-positive tumor cell lines showed that there was stronger development of spots with EpCAM-specific CAR-equipped T cells than with the control T cells transfected with mGFP RNA CAR (Supplementary Figure 4). Correspondingly, analysis with a spot-counting machine indicated that the number of positive spots for anti-EpCAM RNA CAR T cells was significantly higher than for mGFP RNA CAR T cells (*p* < 0.001, Figure 6A, 6B). The findings that anti-EpCAM RNA CAR T cells produced just few number of spots when co-culturing with EpCAM-negative cell lines IGR-1, Raji and PA-1 demonstrated the stimulatory effects of these T cells on IFN-γ secretion in an antigen-specific manner (Figure 6A and Supplementary Figure 4). To ratify that the IFNγ secretion was antigen expression level-dependent, two human colon cancer cell lines, SW480 and HCT8, were transduced with a baculoviral EpCAM-expression vector (BV-EpCAM) or a control BV vector BV-Pax6. Transduction with BV-EpCAM resulted in much higher mean fluorescence intensities of EpCAM expression on the tumor cells (Supplementary Figure 5A). Co-culture of BV-EpCAM transduced tumor cells with anti-EpCAM RNA CAR T cells stimulated more significant IFNγ secretion as compared to the co-cultures of the tumor cells transduced with BV-Pax6 transduced tumors and wildtype tumor cells (Supplementary Figure 5B), indicating an EpCAM expression level-related effect of anti-EpCAM RNA CAR.

We proceeded to determine the antigen-specific cytolytic potential of anti-EpCAM RNA CAR T cells with granzyme B ELISPOT assays. Co-culture of the RNA CAR-modified T cells with EpCAM-positive tumor cell lines HRT-18G and SKOV3-Luc stimulated much higher levels of granzyme B release when compared with co-cultures of mock transfected T cells and T cells transfected with mGFP RNA CAR (Figure 6C).

### Repeated injections of T cells electroporated with mRNA encoding anti-EpCAM CAR delay disease progression in tumor-bearing mice

**Figure 7:**
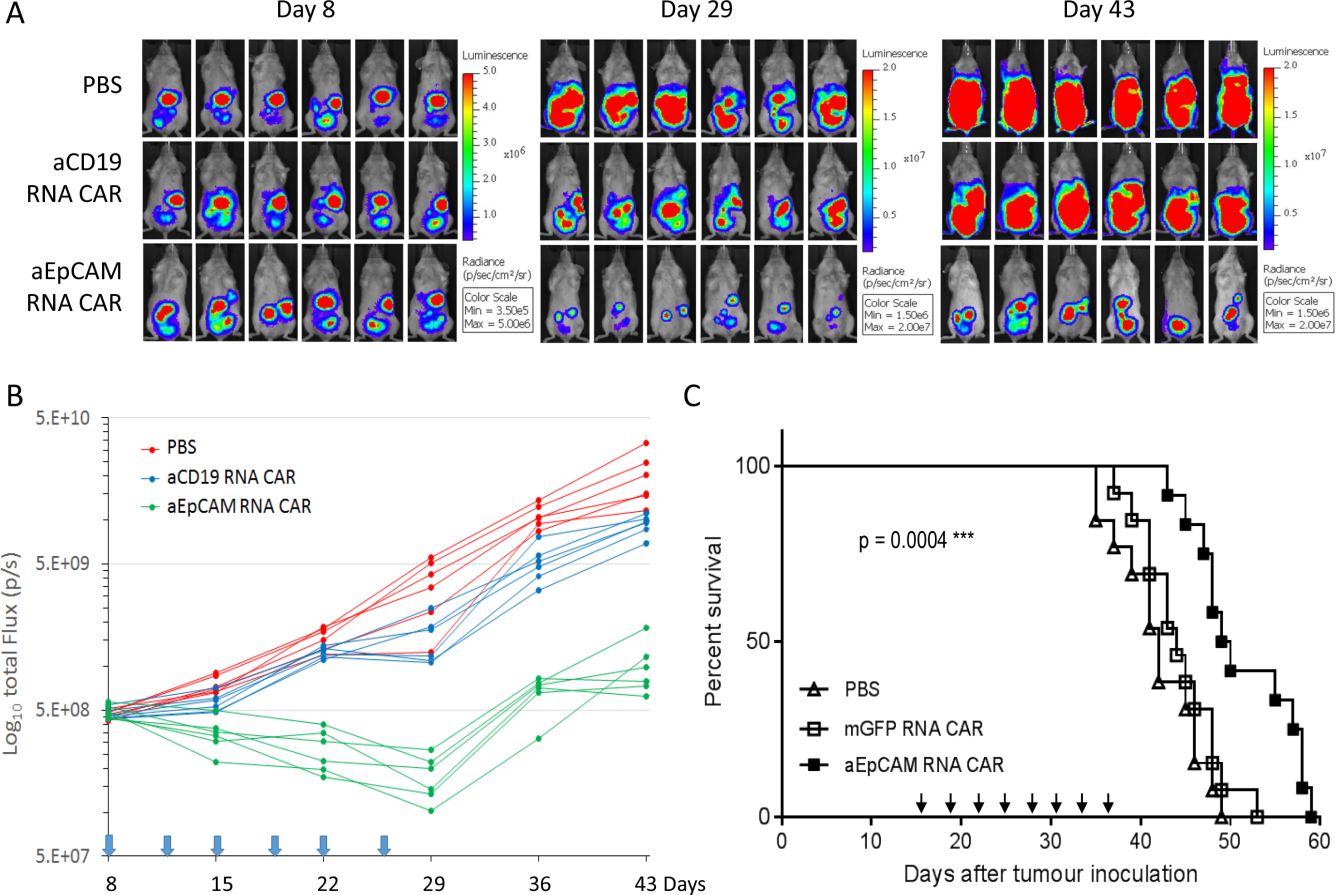
T cells electroporated with anti-EpCAM RNA CAR effectively treat established tumours in NSG mice (**A** and **B**) In mice bearing ovarian tumours. The tumour model was established as described in Figure 4. The mice were randomized into three groups (*n* = 6 per group) before beginning CART cell treatment on day 8. Mice with disseminated ovarian tumours were given multiple doses of 1 × 10^7^ CAR T cells (i.p.) or PBS at the time points indicated by arrows in (**B**). Bioluminescent images prior to treatment (day 8) and following the treatments (days 29, and 43) are shown in (**A**). Tumor burden over time by bioluminescent imaging is shown in (**B**). Each mouse is represented by one line. Kaplan-Meier analysis of survival is shown in Figure 4C. (**C**) In mice bearing colorectal tumours. NSG mice were i.p. injected with 0.5 × 10^7^ human HRT-18G rectal cancer cells on day 0. The mice were randomized into three groups two weeks later. Mice with disseminated colorectal tumours were given multiple doses of 1 × 10^7^ CAR T cells (i.p.) or PBS at the time points indicated by arrows, starting from day 16. This in vivo experiment was repeated two times. Composite survival of two independent experiments (*n* = 13 per group) is shown. Statistical analysis of survival data was performed using the log-rank test.

Encouraged by the *in vitro* results obtained with T cells expressing anti-EpCAM RNA CAR, we next tested their *in vivo* tumor-killing efficiency in the intraperitoneal xenograft mouse model of human ovarian cancer described above. Eight days post-tumor cell injection, the tumor-bearing mice were randomized into three groups (*n* = 6) and i.p injected with PBS, T cells electroporated with an irrelevant anti-CD19 CAR, or T cells electroporated with anti-EpCAM RNA CAR twice a week for 3 weeks. Non-invasive whole-body bioluminescent imaging (BLI) of SKOV3-Luc cells was performed to monitor tumor growth. Figure 7A shows images of mice in each group at 3 different time points post-tumor cell injection. BLI demonstrated that the tumor burden in mice receiving the anti-EpCAM RNA CAR treatment was obviously reduced relative to the initial tumor burdens and that the inhibition of tumour growth by repeated injections of the anti-EpCAM RNA CAR-modified T cells maintained during the 3-week treatment period in all treated mice (Figure 7B). Although tumors regrew after termination of the treatment in mice treated with the anti-EpCAM RNA CAR-modified T cells, most of these mice (5 out of 6) survived for longer than 80 days, significantly longer than mice in the two control groups, which had died or had to be euthanized due to being moribund at days 52–62 (Figure 4C).

We further evaluated the *in vivo* tumor-killing efficiency of mRNA CAR-modified T cells in a xenograft mouse model with established human rectal tumors. The NSG mice were i.p. inoculated with human HRT-18G rectal cancer cells on day 0. Two weeks later, the mice were randomized into three groups for treatment with PBS, mGFP RNA CAR-electroporated T cells, or anti-EpCAM RNA CAR-electroporated T cells. To evaluate the therapeutic effects on late-stage solid tumors, treatment commenced on Day 16 post-tumor inoculation and consisted of eight rounds of i.p. injection of 1E7 CAR T cells or PBS spaced 3 days apart. The experiment was repeated one more time with nearly identical results. The composite survival rate of the two independent experiments is shown in Figure 7C. The median survival time was significantly prolonged from 42 days in the PBS control group and 44 days in the mGFP RNA CAR group to 50 days in the anti-EpCAM RNA CAR group (*p* = 0.0004), demonstrating that repeated injections of anti-EpCAM RNA CAR modified T cells can provide a survival benefit even in an advanced stage of malignancy. While the anti-EpCAM RNA CAR treatment resulted in significant survival advantage, it was unable to achieve disease eradication and animals died or had to be euthanized due to being moribund by day 60. Autopsies were performed on animals that died or were euthanized and extensive intraperitoneal cancer growth was confirmed in all animals (Supplementary Figure 6).

## DISCUSSION

PC often presents in end-stage neoplastic disease and is observed in approximately 70% to 75% of patients with ovarian cancer and 10% to 35% of patients with recurrent colorectal cancer [27]. The combined treatment of PC with cytoreductive surgery and hyperthermic intraperitoneal chemotherapy provides impressive results in patients with PC and has increased chances of patient survival [28, 29]. However, this is a rigorous surgical treatment that carries morbidity and mortality. Also, complete cytoreduction is often not possible and local recurrence still takes place after the treatment in a fraction of patients. As non-operative techniques, intraperitoneal immunotherapy with anti-EpCAM antibody therapeutics has yielded the varying levels of success in treating PC [11, 13, 14]. Nevertheless, there remains an unmet need for more effective treatments for patients with intraperitoneal dissemination of gastrointestinal and gynecological tumors.

We set out to develop an adoptive immunotherapy approach for PC that targets EpCAM-positive tumors and exploits the potent antitumor activity of CAR-expressing T cells. New, third-generation anti-EpCAM CARs were constructed using the scFv 4D5MOCB for CAR targeting. This fragment was derived from the EpCAM-specific mouse monoclonal antibody MOC31 scFv fragment by two modifications: first the antigen binding residues of the MOC31 scFv fragment were grafted onto the humanized framework of the highly stable and well-folding anti-HER-2/neu scFv 4D5 and then eight critical core residues of the heavy chain variable domain core of the parent MOC31 antibody were additionally changed to obtain a high molecule stability [25]. The modified scFV displays similar binding behavior in specificity and in affinity (approximately 3 × 10^-9^ M) comparable to the parent MOC31 scFv fragment [25]. In our CAR design, this humanized anti-EpCAM scFv fragment was fused to the CD8 alpha hinge and transmembrane domain. Intracellularly, the cytoplasmic signaling domains from CD28 and 4-1BB costimulatory receptors were included in tandem with CD3zeta to enhance signal transduction of the CAR and improve T cell persistence.

To transfer our third-generation anti-EpCAM CAR genes into T cells, we tried two different methods, namely lentiviral transduction and electroporation of mRNA, both of which are currently being used in clinical trials. For lentiviral transduction to generate CART cells, we have developed a K562 aAPC-based protocol that can enrich CART cells with improved purity by selectively propagating a small number of stably modified T cells, thus facilitating the use of viral vectors with a relatively lower transduction efficiency and cutting down the cost for viral vector production. To select starting cell populations for viral transduction, we performed pilot experiments to employ the EpCAM CAR-expressing lentiviral vectors to transduce PBMCs activated with CD3/CD28 Dynabeads for 2 days and 7 days. We observed similar transduction efficiencies (15.5 ± 3.22% vs. 15.7 ± 0.45%, *n* = 3) in the 2-day and 7-day PBMC cultures. However, after 21 days of co-culturing with K562A-EpCAM aAPCs, the difference in CART cell enrichment was rather noticeable: 40% of CAR-positive T cells were obtained when the 2-day PBMC cultures were used for transduction, whereas > 80% of CAR-positive T cells were obtained when PBMCs activated for 7 days were used for the transduction (Figure 2C). Further characterization of the phenotypes of expanded immune cells after the K562 co-culture revealed much higher percentages of CD3-CD56+ NK cells derived from the 2-day PBMC cultures: 44.9 ± 5.29% in the 2-day PBMC cultures vs. 5.8 ± 2.86% the 7-day PBMC cultures (*n* = 3). This is possibly because the treatment of PBMCs with CD3/CD28 Dynabeads for 2 days was not enough to generate pure T cell populations as the protocol of 7-day Dynabead activation does. To test pure CART cells, we have adopted the 7-day activation protocol in the current study.

One of the important findings from this study is delayed disease progression in tumor-bearing mice by repeated injections of T cells with transient expression of anti-EpCAM CAR by mRNA electroporation. The therapeutic effects were observed even in mice with well-established peritoneal xenografts (tumor cells inoculated 16 days ago). As demonstrated by BLI, the repeated treatment could reduce tumor burden and sustain the tumor growth inhibitory effects in all treated mice during the whole treatment period (Figure 7B). This finding suggests an interesting therapy approach for disease reduction in cancer patients by regular, repeated injections of T cells transfected with mRNA encoding an EpCAM-targeting CAR. The approach is attractive given that the self-limited expression of CAR following transient mRNA transfection may be an advantage in terms of safety.

We have attempted to improve the stability of mRNA encoding the CAR by including α-globin 3′ untranslated region, a prolonged (150A) poly(**A**) sequence, and capping with an anti-reverse cap analogue or the cap1 structure [30]. We injected the CAR-modified T cells intraperitoneally in mouse PC models. Intraperitoneal administration offers the benefit of increasing local concentration of effector cells to target tumor cells or tumor cell clusters distributed on an extended surface of the peritoneum, while minimizing systemic adverse effects by limiting the systemic distribution of the cells in the body. The localized immunological processes in the abdominal cavity may also contribute to mitigating unwanted systemic immune reactions.

Although EpCAM is attractive for targeted cancer therapy, the fact that EpCAM is expressed also on normal epithelium, though to a much lower level, is a concern for potential on-target/off-tumor toxicity of targeted therapy against EpCAM-positive tumors. Obviously, potent anticancer immunotherapy approaches based on CAR are faced with even more challenges in controlling on-target/off-tumor toxicity and cytokine storms. Strategies involving the use of mRNA CARs deserve consideration in this aspect [30, 31]. Rather than transducing T cells with a viral vector mediating permanent CAR expression that cannot be simply shut off if toxic, mRNA CAR approaches have been developed by generating short-lived CAR-expressing cells through mRNA transfection to reduce the duration and potency of CAR effects, thus decreasing potential toxicity. Understandably, the transient expression of CARs on T cells requires repeated infusions to achieve antitumor effects. It, however, provides good opportunity to test CAR clinical safety. Through discontinuing the infusion of mRNA CART cells, an excessive response caused by the toxicity related to recognition of normal tissues and/or cytokine storms can be controlled. Transduction of T cells with a viral vector for CAR gene expression also comes with practical constraints, such as being expensive and laborious for large-scale virus manufacturing in compliance with cGMP. Transfection of mRNA encoding a CAR could be more economical in facilitating the design and testing of new CARs, particularly when performing an initial clinical trial of a novel CAR.

In preclinical models, multiple infusions of mRNA CAR-modified T cells have shown potent anti-tumor effects [30, 31–37]. Whether intraperitoneal administration of EpCAM-specific CAR-modified T cells, especially those modified through mRNA electroporation, will be safe and effective warrants further preclinical and clinical evaluation.

## MATERIALS AND METHODS

### Tumor cell lines

Human ovarian cancer cell line SKOV3-Luciferase (SKOV3-Luc, PerkinElmer, Waltham, MA) was maintained in McCoy's 5A supplemented with 800 μg/ml G418 and 10% heat-inactivated fetal bovine serum (FBS, Hyclone, Logan, UT). Human melanoma cell line IGR1 (from DSMZ, Braunschweig, Germany) were cultured in DMEM supplemented with 10% FBS. Human ovarian cancer cell lines PA-1, SW626, and CAOV3 and human colorectal adenocarcinoma cell lines HCT8, HRT-18G, SW480, and SW620, and human myelogenous leukemia cell line K562 were purchased from ATCC (Manassas, VA) and maintain as instructed.

### DNA constructs for aAPC generation

To construct a plasmid vector for the generation of human myelogenous leukemia cell line K562-based aAPCs for CART cell expansion, the gene encoding sequences for CD64 (FcγRI, GenBank accession no. BC032634), CD86 (B7-2, GenBank accession no. NM_175862) and CD137L (4-1BBL, GenBank accession no. NM_003811) were PCR-amplified from a human PBMC cDNA library, respectively, and subsequently cloned into pFastBac1-CMV-EGFP vector to generate the costimulatory molecule vector as shown in Figure 1A. K562 cells were transfected with the plasmid vector and selected with 1 μg/ml puromycin (Life Technologies, Carlsbad, CA). Single-cell cloning was performed and one clone, named K562A, with the highest expression of CD64, 41BB and CD86 was selected. The extracellular part of EpCAM (uniprot P16422, residues 1-288) gene was amplified from the colon cancer cell line HCT8 cDNA library, and subcloned into an AAVS1 donor vector [38] to generate the EpCAM vector. This vector was then used as the DNA donor vector and co-transfected together with a zinc-finger nuclease (ZFN) vector [46] for AAVS1 locus-specific gene insertion. After puromycin (1 μg/ml) and G418 (500 μg/ml) selection for three weeks, single cell sorting, and EpCAM expression analysis, a single-cell clone with the highest EpCAM expression was selected and named K562A-EpCAM. To prepare K562A-EpCAM aAPCs for CAR T cell expansion experiments, the cells were γ-irradiated (100 Gy for 100 min) and cryopreserved.

### Anti-EpCAM CAR lentiviral vector and generation of CART cells stably expressing the CAR

The anti-EpCAM CAR sequence was designed by in silico assembly of fragments encoding the single-chain variable fragment (scFv) from 4D5MOC-B humanized mAb [28], the CD8 alpha hinge and transmembrane domain (uniprot P01732, residues 128-210), CD28 co-stimulatory domain (uniprot P10747, residues 180–220), 4-1BB co-stimulatory domain (uniprot Q07011, residues 214–255), and the CD3zeta intracellular ITAM domain (uniprot P20963,residues 52–164), followed by synthesis of the codon-optimized fusion gene (AIT Biotech, Singapore). The complete anti-EpCAM CAR sequence was inserted into the third generation self-inactivating lentiviral expression vector (CD810A-1; System Biosciences, Mountain View, CA) after EcoRI and SalI digestion. A control vector was produced by replacing the anti-EpCAM scFv with the GFP coding sequence. Lentiviruses were produced in HEK293FT cells (Life Technologies). The viral supernatant was harvested and concentrated through ultracentrifugation for 3 hours at 25,000 rpm.

To generate lentivirus-transduced CART cells, human peripheral blood mononuclear cells (PBMCs) were isolated from fresh buffy coat of healthy donors by density gradient centrifugation using Ficoll-Paque (GE Healthcare, Milwaukee, WI). The isolated PBMCs were activated using human CD3/CD28 dynabeads (Life Technologies) in AIM-V media (Life Technologies) supplemented with 5% inactivated human AB serum (Valley Biomedical, Winchester, VA), followed by culturing in the presence of 300 IU/ml IL-2 (Peprotech, Rocky Hill, NJ), 5 ng/ml IL-7 (Peprotech), and 5 ng/ml IL-15 (Peprotech) human cytokines. After one week of culture, T cells were transduced with either anti-EpCAM CAR- or mGFP CAR-lentivirus supernatant. One week after transduction, T cells expressing anti-EpCAM CAR were selectively expanded by co-culturing with γ-irradiated K562A-EpCAM aAPCs at a ratio of 1:2. To expand T cells expressing mGFP CAR, FACS was used to enrich the GFP CAR-expressing T cells, followed by co-culture with γ-irradiated K562A-EpCAM at a ratio of 1:50 with IL-2, IL-7 and IL-15.

### Anti-EpCAM mRNA CAR and electroporation of T cells

To generate anti-EpCAM mRNA CAR vectors, the above anti-EpCAM CAR sequence was subcloned into a pFastbac1 (Life Technologies) with EcoRI and SalI. For *in vitro* transcription of mRNA, the pFastbac1 vector was modified by adding a T7 promoter, a 5′UTR with Kozak sequence, a GM-CSF signal peptide (SP) encoding sequence and an alpha-globin 3′UTR sequence [39]. PCR was performed using the pFastbac1 vector as the DNA template, a forward primer CMV-F (5′-atccgctcgagtagttattaatagtaatcaattacggggtc-3′), and reverse primer T150-R. The control vector anti-CD19 CAR was produced by replacing the anti-EpCAM scFv with an anti-CD19 scFv sequence. Capped mRNA was generated through *in vitro* transcription of the PCR DNA templates using the mMESSAGE mMACHINE T7 ULTRA transcription kit (Invitrogen, Carlsbad, CA) or the mScript™ RNA system (Epicentre, Madison, WI). For mRNA electroporation, 0.2 ml of the expanded T cells was mixed with 20 μg mRNA and electroporated in a 2-mm cuvette (Bio-Rad, Hercules, CA) using a NEPA21 electroporator (Nepagene, Chiba, Japan) with the following parameters: voltage 240 V, pulse length 4 ms, pulse once. The electroporated T cells were rested for 3 hours and frozen to −80°C until injection.

### Flow cytometric analysis, Western blot analysis, and ELISPOT assays

Cell surface expression of CAR was detected using biotin-SP (long spacer) AffiniPure F(ab’)_2_ fragment goat anti-mouse IgG (115-066-072; Jackson Immunoresearch Laboratories, West Grove, PA) for 25 minutes, followed by allophycocyanin-conjugated streptavidin (016-130-084; Jackson). For phenotypic analysis of T cells, the following conjugated anti-human antibodies were used: CD3 (clone: OKT3; eBioscience, San Diego, CA), CD45RA (clone: HI100; eBioscience), and CCR7 (clone: 3D12; eBioscience). The following antibodies were used for staining of cell lines: CD64, CD86, CD137L, and EpCAM (clone: HEA-125; Miltenyi, Bergisch Gladbach, Germany). For staining, cells were re-suspended in FACS buffer (PBS, 2% FBS, 0.1% sodium azide) and stained with antibodies in dark at 4°C for 30 minutes. The cells were washed twice with the MACS buffer (Miltenyi) prior to and post-staining. Appropriate isotype controls were used to validate gating.

For Western blot analysis to detect CAR expression, cells were lysed in RIPA lysis and extraction buffer (Santa Cruz Biotechnology, Santa Cruz, CA) supplemented with protease inhibitor (Roche Diagnostics, Pleasanton, CA) and phenylmethyl sulfonyl fluoride (Sigma-Aldrich). Proteins in the lysis solution (60 μg) were separated in 10% acrylamide gradient gel and transferred to nitrocellulose membrane. The membrane was probed with rabbit anti-human anti-CD3 zeta antibody (HPA008750; 1:1000 diluted, Sigma-Aldrich) overnight at 4°C. The washed membrane was incubated with goat anti-rabbit or goat anti-mouse secondary antibody (1:5000 diluted, Santa Cruze Biotech) for 1 hour at room temperature, followed by development and visualization.

IFNγ secretion and granzyme B up-regulation triggered by tumor antigen specific recognition of anti-EpCAM CARs was determined by IFNγ and granzyme B ELISPOT assays according to the protocols of ELISPOT kits (Mabtech, Nacka Strand, Sweden). The plates were analyzed by an ELISPOT scanner (CTL, Ltd., of. Cleveland, OH).

### Cytotoxicity assay

The cytolytic activity of CAR-modified T cells was examined with the DELFIA EuTDA Cytotoxicity Reagents kit (PerkinElmer). The effector to target (E:T) ratios used ranged from 40:1 to 1:1. Control groups were set up to measure spontaneous release (only target cells added), maximum release (target cells added with 10 μl lysis buffer), and medium background (no cell added). Killing efficacy was calculated by using the following formula:

$$\text{\% Specific release}=\frac{\text{Experimental release (counts) -Spontaneous release (counts)}}{\text{Maximum release (counts) -Spontaneous release (counts)}}\times 100$$

### Animal experiments

Non-obese diabetic/severe combined immuno- deficiency/IL-2Rγcnull (NSG) mice (6–8 weeks old, female) were used in the current study. A mouse xenograft model of human ovarian carcinoma was established by inoculating via intraperitoneal (i.p.) injection of 1 × 10^7^ SKOV3-Luc cells. On day 8 post-tumor inoculation, tumor engraftment was confirmed by live bioluminescence imaging (BLI) monitored using an IVIS Spectrum Imaging platform with Living Image software (PerkinElmer). Mice with similar BLI signal intensity randomly divided into 3 different treatment groups, 6 mice per group. Injections of phosphate-buffered saline (PBS), mGFP CAR T cells or T cells electroporated with an irrelevant anti-CD19 CAR, a CAR construct with a non-functional scFv targeting an irrelevant antigen, served as negative controls. EpCAM-specific DNA and RNA CAR T cells were prepared by lentiviral transduction and electroporation, respectively. A single dose of 10 million CAR-modified T cells in 100 μl of PBS or the same volume of PBS without cells was i.p. injected on day 8 to test the *in vivo* anti-tumor activity of anti-EpCAM DNA CAR. To test the *in vivo* anti-tumor activity of anti-EpCAM RNA CAR, the treatment started 8 days after tumor cell inoculation, twice a week for 3 weeks, with 10 million CAR-modified T cells in 100 μl of PBS or the same volume of PBS without cells per injection. Tumor progression was monitored by BLI. All luminescent signals and images were acquired and analyzed with the Xenogen living imaging software v2.5.

A mouse xenograft model of human colorectal carcinoma was also established by inoculating via intraperitoneal (i.p.) injection of 0.5 × 10^7^ HRT-18G cells. Two weeks later, mice were randomly divided into 3 different treatment groups, i.e. PBS, mGFP RNA CAR T cell, and anti-EpCAM RNA CAR T cell groups, 8 mice per group. The treatment started at day 16 post-tumor inoculation in order to evaluate the therapeutic effects on late-stage solid tumors. Mice were i.p. injected with 1E7 CAR T cells or PBS eight rounds spaced 3 days apart. This experiment was repeated again with 5 mice per group.

Mice were monitored closely and humanely euthanized after observing the development of moribund condition characterized by obvious abdominal bloating due to ascites, palpable hypothermia, inability to walk, and/or lack of overt response to manipulation. The treated mice with SKOV3-Luc xenografts were euthanized at day 80.

### Statistical analysis

All data are presented as mean ± SD. Differences between values were evaluated using the two tailed unpaired Student's *t* test. Differences between groups were evaluated by the two-factor analysis of variance with replication followed by Fisher's least significant difference post hoc analysis. For survival studies, Kaplan-Meier survival curves were plotted and analyzed using Prism software (GraphPad Software). *p* values < 0.05 were considered significant.

### Ethics statement

The use of fresh buffy coats of healthy donors for human PBMC isolation was approved by the institutional review board of National University of Singapore (NUS-IRB Reference Code B-14-133E) based on the fact that the research uses only anonymous buff coats/apheresis ring belt from the National University Hospital, Department of Laboratory Medicine Blood Transfusion Service.

All handling and care of animals was performed according to the guidelines for the Care and Use of Animals for Scientific Purposes issued by the National Advisory Committee for Laboratory Animal Research, Singapore. The animal study protocol was reviewed and approved by Institutional Animal Care and Use Committee (IACUC), the Biological Resource Centre, the Agency for Science, Technology and Research (A*STAR), Singapore (Permit Number: BRC IACUC 110612).

## SUPPLEMENTARY MATERIALS FIGURES AND TABLES



## References

[1] Mellstedt H, Fagerberg J, Frödin JE, Hjelm-Skog AL, Liljefors M, Markovic K, Mosolits S, Ragnhammar P (2000). Ga733/EpCAM as a target for passive and active specific immunotherapy in patients with colorectal carcinoma. Ann N Y Acad Sci.

[2] Schnell U, Cirulli V, Giepmans BNG (2013). EpCAM: structure and function in health and disease. Biochim Biophys Acta.

[3] Simon M, Stefan N, Plückthun A, Zangemeister-Wittke U (2013). Epithelial cell adhesion molecule-targeted drug delivery for cancer therapy. Expert Opin Drug Deliv.

[4] Martowicz A, Seeber A, Untergasser G (2016). The role of EpCAM in physiology and pathology of the epithelium. Histol Histopathol.

[5] Trzpis M, McLaughlin PMJ, de Leij LMFH, Harmsen MC (2007). Epithelial cell adhesion molecule: more than a carcinoma marker and adhesion molecule. Am J Pathol.

[6] Patriarca C, Macchi RM, Marschner AK, Mellstedt H (2012). Epithelial cell adhesion molecule expression (CD326) in cancer: a short review. Cancer Treat Rev.

[7] Maetzel D, Denzel S, Mack B, Canis M, Went P, Benk M, Kieu C, Papior P, Baeuerle PA, Munz M, Gires O (2009). Nuclear signalling by tumour-associated antigen EpCAM. Nat Cell Biol.

[8] Balzar M, Winter MJ, de Boer CJ, Litvinov SV (1999). The biology of the 17-1A antigen (Ep-CAM). J Mol Med Berl Ger.

[9] Di Paolo C, Willuda J, Kubetzko S, Lauffer I, Tschudi D, Waibel R, Plückthun A, Stahel RA, Zangemeister-Wittke U (2003). A recombinant immunotoxin derived from a humanized epithelial cell adhesion molecule-specific single-chain antibody fragment has potent and selective antitumor activity. Clin Cancer Res Off J Am Assoc Cancer Res.

[10] Munz M, Baeuerle PA, Gires O (2009). The emerging role of EpCAM in cancer and stem cell signaling. Cancer Res.

[11] Heiss MM, Murawa P, Koralewski P, Kutarska E, Kolesnik OO, Ivanchenko VV, Dudnichenko AS, Aleknaviciene B, Razbadauskas A, Gore M, Ganea-Motan E, Ciuleanu T, Wimberger P (2010). The trifunctional antibody catumaxomab for the treatment of malignant ascites due to epithelial cancer: Results of a prospective randomized phase II/III trial. Int J Cancer J Int Cancer.

[12] Linke R, Klein A, Seimetz D (2010). Catumaxomab: clinical development and future directions. mAbs.

[13] Ströhlein MA, Lordick F, Rüttinger D, Grützner KU, Schemanski OC, Jäger M, Lindhofer H, Hennig M, Jauch KW, Peschel C, Heiss MM (2011). Immunotherapy of peritoneal carcinomatosis with the antibody catumaxomab in colon, gastric, or pancreatic cancer: an open-label, multicenter, phase I/II trial. Onkologie.

[14] Berek JS, Edwards RP, Parker LP, DeMars LR, Herzog TJ, Lentz SS, Morris RT, Akerley WL, Holloway RW, Method MW, Plaxe SC, Walker JL, Friccius-Quecke H (2014). Catumaxomab for the treatment of malignant ascites in patients with chemotherapy-refractory ovarian cancer: a phase II study. Int J Gynecol Cancer Off J Int Gynecol Cancer Soc.

[15] MacDonald GC, Rasamoelisolo M, Entwistle J, Cizeau J, Bosc D, Cuthbert W, Kowalski M, Spearman M, Glover N (2009). A phase I clinical study of VB4-845: Weekly intratumoral administration of an anti-EpCAM recombinant fusion protein in patients with squamous cell carcinoma of the head and neck. Drug Des Devel Ther.

[16] Kowalski M, Entwistle J, Cizeau J, Niforos D, Loewen S, Chapman W, MacDonald GC (2010). A Phase I study of an intravesically administered immunotoxin targeting EpCAM for the treatment of nonmuscle-invasive bladder cancer in BCGrefractory and BCG-intolerant patients. Drug Des Devel Ther.

[17] Andersson Y, Engebraaten O, Juell S, Aamdal S, Brunsvig P, Ø Fodstad, Dueland S (2015). Phase I trial of EpCAM-targeting immunotoxin MOC31PE, alone and in combination with cyclosporin. Br J Cancer.

[18] Tavri S, Jha P, Meier R, Henning TD, Müller T, Hostetter D, Knopp C, Johansson M, Reinhart V, Boddington S, Sista A, Wels WS, Daldrup-Link HE (2009). Optical imaging of cellular immunotherapy against prostate cancer. Mol Imaging.

[19] Meier R, Golovko D, Tavri S, Henning TD, Knopp C, Piontek G, Rudelius M, Heinrich P, Wels WS, Daldrup-Link H (2011). Depicting adoptive immunotherapy for prostate cancer in an animal model with magnetic resonance imaging. Magn Reson Med.

[20] Sahm C, Schönfeld K, Wels WS (2012). Expression of IL-15 in NK cells results in rapid enrichment and selective cytotoxicity of gene-modified effectors that carry a tumor-specific antigen receptor. Cancer Immunol Immunother.

[21] Shirasu N, Yamada H, Shibaguchi H, Kuroki M, Kuroki M, Shirasu N, Yamada H, Shibaguchi H, Kuroki M, Kuroki M (2012). Molecular Characterization of a Fully Human Chimeric T-Cell Antigen Receptor for Tumor-Associated Antigen EpCAM, Molecular Characterization of a Fully Human Chimeric T-Cell Antigen Receptor for Tumor-Associated Antigen EpCAM. BioMed Res Int BioMed Res Int.

[22] Shirasu N, Yamada H, Shibaguchi H, Kuroki M, Kuroki M, Shirasu N, Yamada H, Shibaguchi H, Kuroki M, Kuroki M (2015). Corrigendum to “Molecular Characterization of a Fully Human Chimeric T-Cell. Antigen Receptor for Tumor-Associated Antigen EpCAM.” BioMed Res Int BioMed Res Int.

[23] Deng Z, Wu Y, Ma W, Zhang S, Zhang Y-Q (2015). Adoptive T-cell therapy of prostate cancer targeting the cancer stem cell antigen EpCAM. BMC Immunol.

[24] Wu Y, Deng Z, Tang Y, Zhang S, Zhang Y-Q (2015). Over-expressing Akt in T cells to resist tumor immunosuppression and increase anti-tumor activity. BMC Cancer.

[25] Willuda J, Honegger A, Waibel R, Schubiger PA, Stahel R, Zangemeister-Wittke U, Plückthun A (1999). High thermal stability is essential for tumor targeting of antibody fragments: engineering of a humanized anti-epithelial glycoprotein-2 (epithelial cell adhesion molecule) single-chain Fv fragment. Cancer Res.

[26] Spizzo G, Fong D, Wurm M, Ensinger C, Obrist P, Hofer C, Mazzoleni G, Gastl G, Went P (2011). EpCAM expression in primary tumour tissues and metastases: an immunohistochemical analysis. J Clin Pathol.

[27] Coccolini F, Gheza F, Lotti M, Virzì S, Iusco D, Ghermandi C, Melotti R, Baiocchi G, Giulini SM, Ansaloni L, Catena F (2013). Peritoneal carcinomatosis. World J Gastroenterol.

[28] Yonemura Y, Canbay E, Endou Y, Ishibashi H, Mizumoto A, Miura M, Li Y, Liu Y, Takeshita K, Ichinose M, Takao N, Hirano M, Sako S (2014). Peritoneal cancer treatment. Expert Opin Pharmacother.

[29] Guend H, Patel S, Nash GM (2015). Abdominal metastases from colorectal cancer: intraperitoneal therapy. J Gastrointest Oncol.

[30] Zhao Y, Moon E, Carpenito C, Paulos CM, Liu X, Brennan AL, Chew A, Carroll RG, Scholler J, Levine BL, Albelda SM, June CH (2010). Multiple injections of electroporated autologous T cells expressing a chimeric antigen receptor mediate regression of human disseminated tumor. Cancer Res.

[31] Beatty GL, Haas AR, Maus MV, Torigian DA, Soulen MC, Plesa G, Chew A, Zhao Y, Levine BL, Albelda SM, Kalos M, June CH (2014). Mesothelin-specific chimeric antigen receptor mRNA-engineered T cells induce anti-tumor activity in solid malignancies. Cancer Immunol Res.

[32] Barrett DM, Zhao Y, Liu X, Jiang S, Carpenito C, Kalos M, Carroll RG, June CH, Grupp SA (2011). Treatment of advanced leukemia in mice with mRNA engineered T cells. Hum Gene Ther.

[33] Barrett DM, Liu X, Jiang S, June CH, Grupp SA, Zhao Y (2013). Regimen-specific effects of RNA-modified chimeric antigen receptor T cells in mice with advanced leukemia. Hum Gene Ther.

[34] Singh N, Barrett DM, Grupp SA (2014). Roadblocks to success for RNA CARs in solid tumors. Oncoimmunology.

[35] Singh N, Liu X, Hulitt J, Jiang S, June CH, Grupp SA, Barrett DM, Zhao Y (2014). Nature of tumor control by permanently and transiently modified GD2 chimeric antigen receptor T cells in xenograft models of neuroblastoma. Cancer Immunol Res.

[36] Kenderian SS, Ruella M, Shestova O, Klichinsky M, Aikawa V, Morrissette JJD, Scholler J, Song D, Porter DL, Carroll M, June CH, Gill S (2015). CD33-specific chimeric antigen receptor T cells exhibit potent preclinical activity against human acute myeloid leukemia. Leukemia.

[37] Schutsky K, Song DG, Lynn R, Smith JB, Poussin M, Figini M, Zhao Y, Powell DJ (2015). Rigorous optimization and validation of potent RNA CAR T cell therapy for the treatment of common epithelial cancers expressing folate receptor. Oncotarget.

[38] Phang RZ, Tay FC, Goh SL, Lau CH, Zhu H, Tan WK, Liang Q, Chen C, Du S, Li Z, Tay JC, Wu C, Zeng J (2013). Zinc finger nuclease-expressing baculoviral vectors mediate targeted genome integration of reprogramming factor genes to facilitate the generation of human induced pluripotent stem cells. Stem Cells Transl Med.

[39] Warren L, Manos PD, Ahfeldt T, Loh Y-H, Li H, Lau F, Ebina W, Mandal PK, Smith ZD, Meissner A (2010). Highly efficient reprogramming to pluripotency and directed differentiation of human cells with synthetic modified mRNA. Cell Stem Cell.

