# Supplementary material for: Intraperitoneal immunotherapy with T cells stably and transiently expressing anti-EpCAM CAR in xenograft models of peritoneal carcinomatosis
Source: Oncotarget. 2017 Jan 10;8(8):13545–59. doi: 10.18632/oncotarget.14592 (PMC5355119; doi:10.18632/oncotarget.14592)
Supplement: Supplementary file 1 [file oncotarget-08-13545-s001.pdf]

Intraperitoneal immunotherapy with T cells stably and transiently expressing anti-EpCAM cAR in xenograft models of peritoneal carcinomatosis

Supplementary Materials

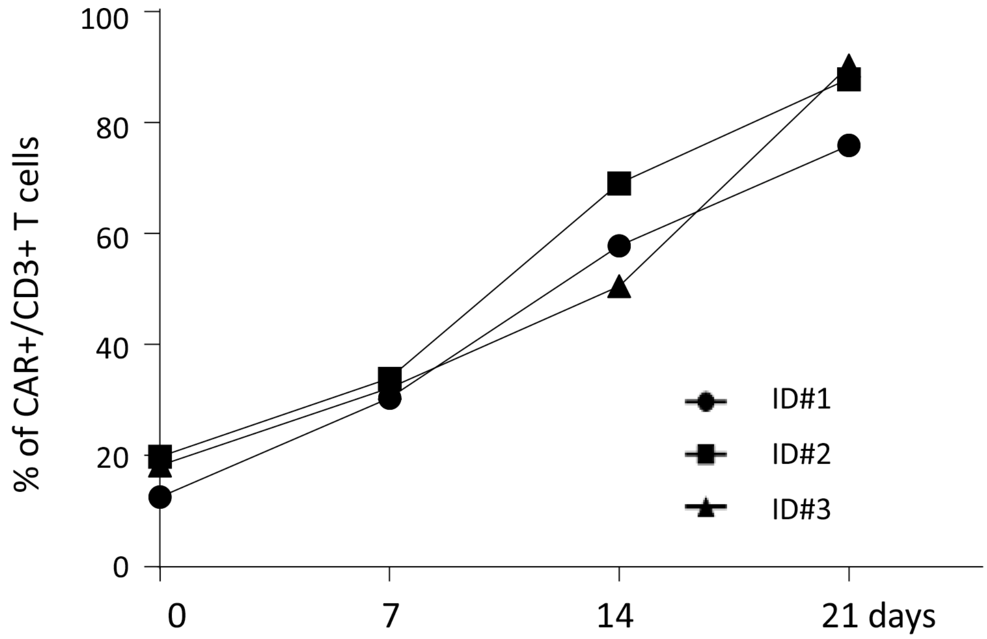

Supplementary Figure 1: Expansion of EpCAM-specific CART cells. CD3+CAR+ T cells increase over the co-culturing with K562A-EpCAM aAPCs. Results from three different PBMC samples are shown.

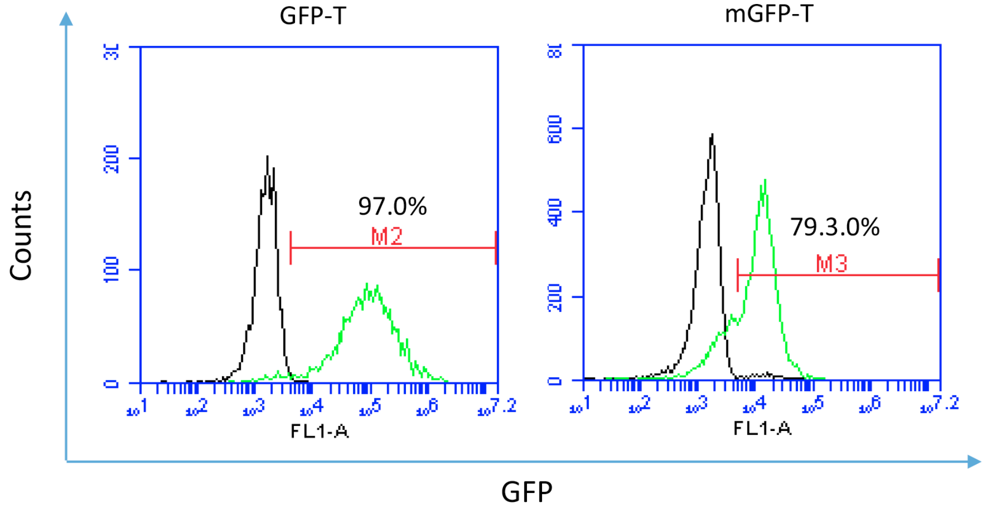

Supplementary Figure 2: The electroporation efficiency in T cells. The cells were transfected with normal GFP mRNA for cytoplasm expression and mGFPmRNA CAR for membrane expression. GFP expression was examined by flow cytometry.

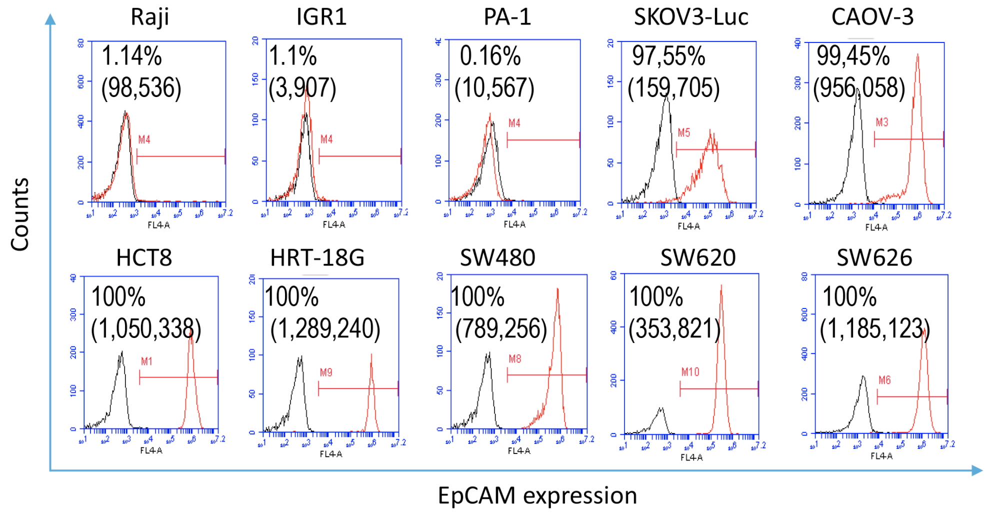

Supplementary Figure 3: EpCAMexpression in various types of tumor cell lines. Human lymphoma line Raji, melanoma line IGR1, ovarian cancer lines PA-1, SKOV3-Luc and CAOV3, and colorectal cancer lines HCT8, HRT-18G, SW480, SW620 and SW626 were tested. Percentage positive cells (%) and mean fluorescence intensity (MFI) values are shown.

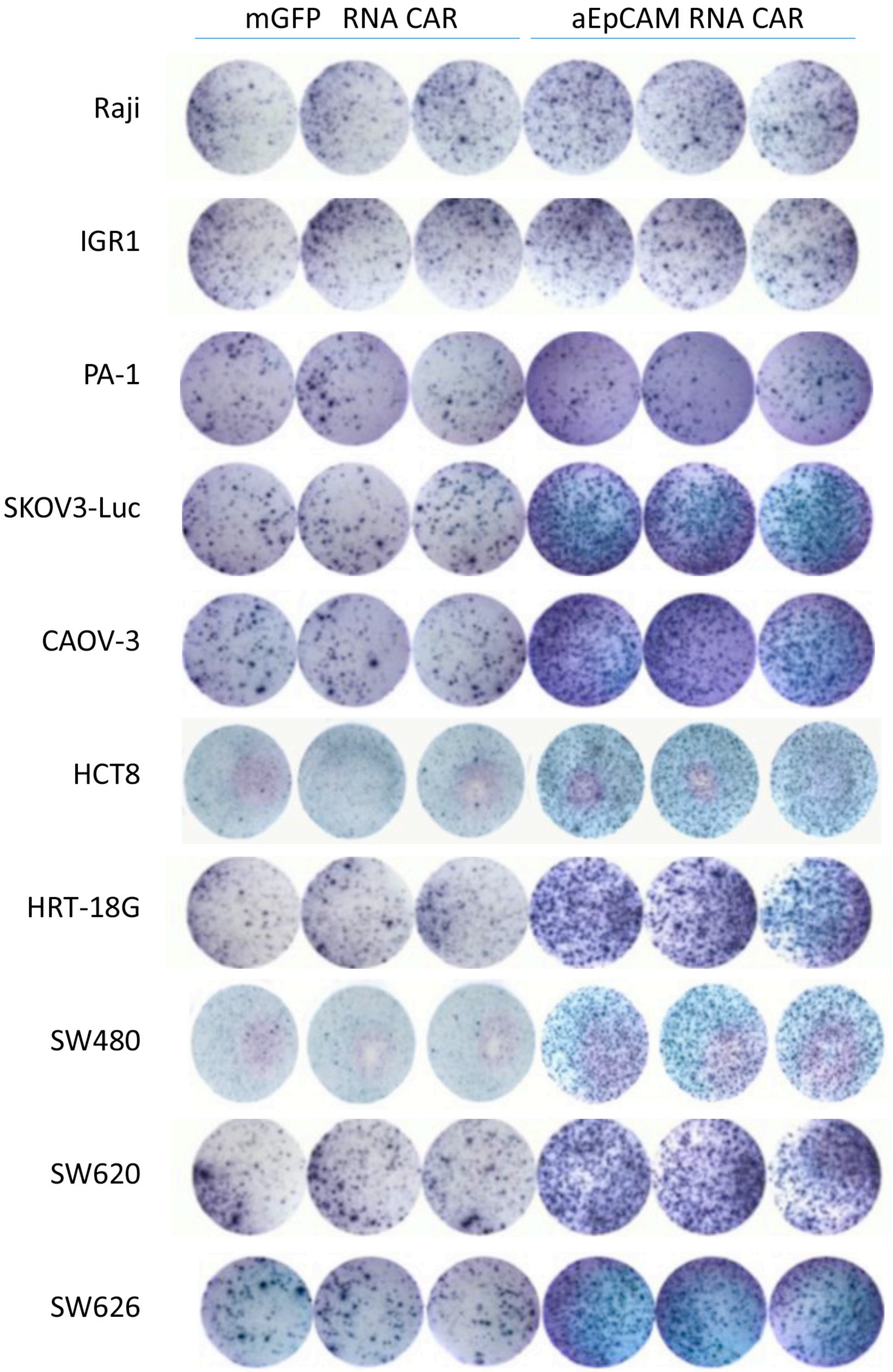

Supplementary Figure 4: IFNγ secretion triggered by tumor antigen-specific recognition of anti-EpCAM RNA CARs as determined by IFNγ ELISPOT assay. T cells were electroporated with mGFP RNA CAR or EpCAM-specific RNA CAR and co-cultured with indicated target tumor cells overnight before ELISPOT assay. Images from the plate scan of the ELISPOT assay are shown.

A

Cell line_BV Transduction	EpCAM%	EpCAM MFI
SW480 WT	100%	789,256
SW480_BV_Pax6	100%	1,076,844
SW480_BV_EpCAM	100%	1,778,613
HCT8 WT	100%	1,050,338
HCT8_BV_pax6	100%	1,139,722
HCT8_BV_EpCAM	100%	1,481,640

B

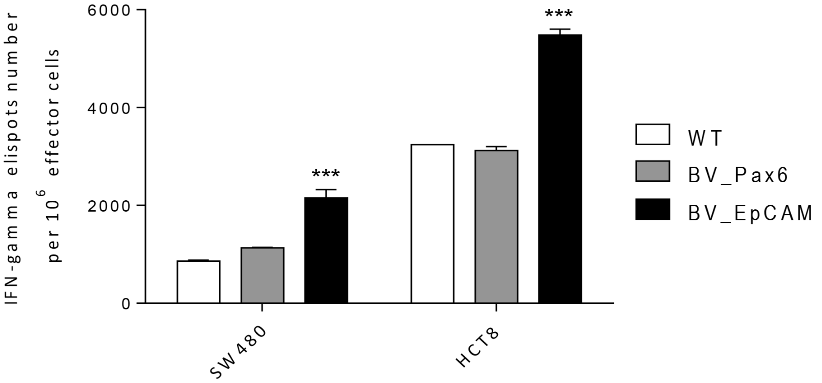

Supplementary Figure 5: Increased IFNγ secretion after transduction of tumor cells with a baculoviral vector expressing EpCAM (BV-EpCAM). (**A**) Up-regulation of EpCAM expression on colon cancer cells by baculoviral transduction. Two human colon cancer cell lines, SW480 and HCT8, were transduced with a baculovirus EpCAM-expression vector (BV-EpCAM) or a control BV vector BVPax6. Transduction with BV-EpCAM did not change the percentage of EpCAM expression but resulted in much higher mean fluorescence intensities of EpCAM expression on the tumor cells. (**B**) IFNγ secretion. One day after transduction, the tumor cells were mixed with T cells electroporated with anti- EpCAM mRNA CARs and co-cultured overnight before IFNγ ELISPOT assay.

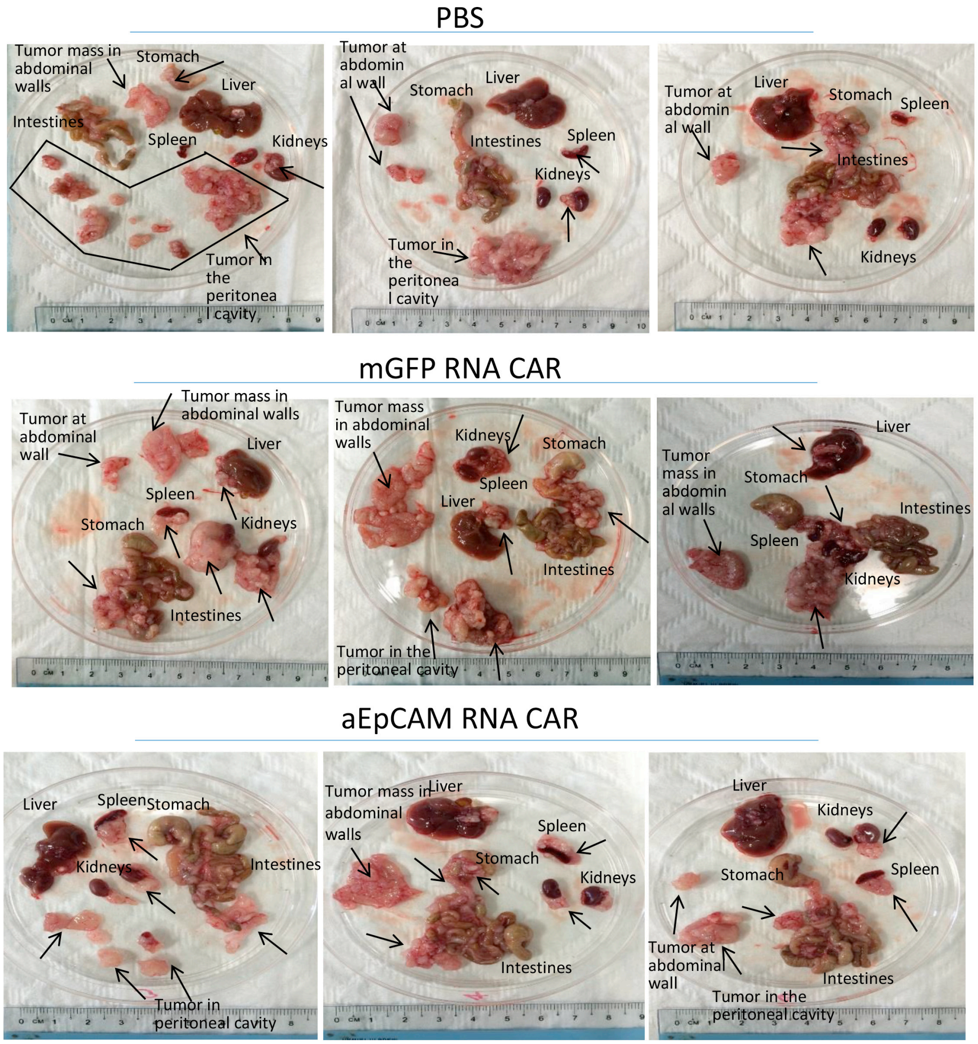

Supplementary Figure 6: Autopsy of the euthanized animals tested in Figure 7C. Large tumors scattered all over the peritoneal cavity were found, surrounding the pancreatic area, spleen, kidneys, liver and intestines. Masses of necrotic tissues in the abdominal wall were also noticed some of the euthanizedmice. Samples from 3 mice per group are shown.
